# Supplementary material for: Splice-Junction-Based Mapping of Alternative Isoforms in the Human Proteome
Source: Cell Rep. Author manuscript; Available in PMC 2020 Jan 15. (PMC6961840; doi:10.1016/j.celrep.2019.11.026)
Supplement: 2 [file NIHMS1546469-supplement-2.zip › DF1/PXD009737/TestisGluC/TestisGluC_15_RANBP1_DAQKFKTKFE.pdf]

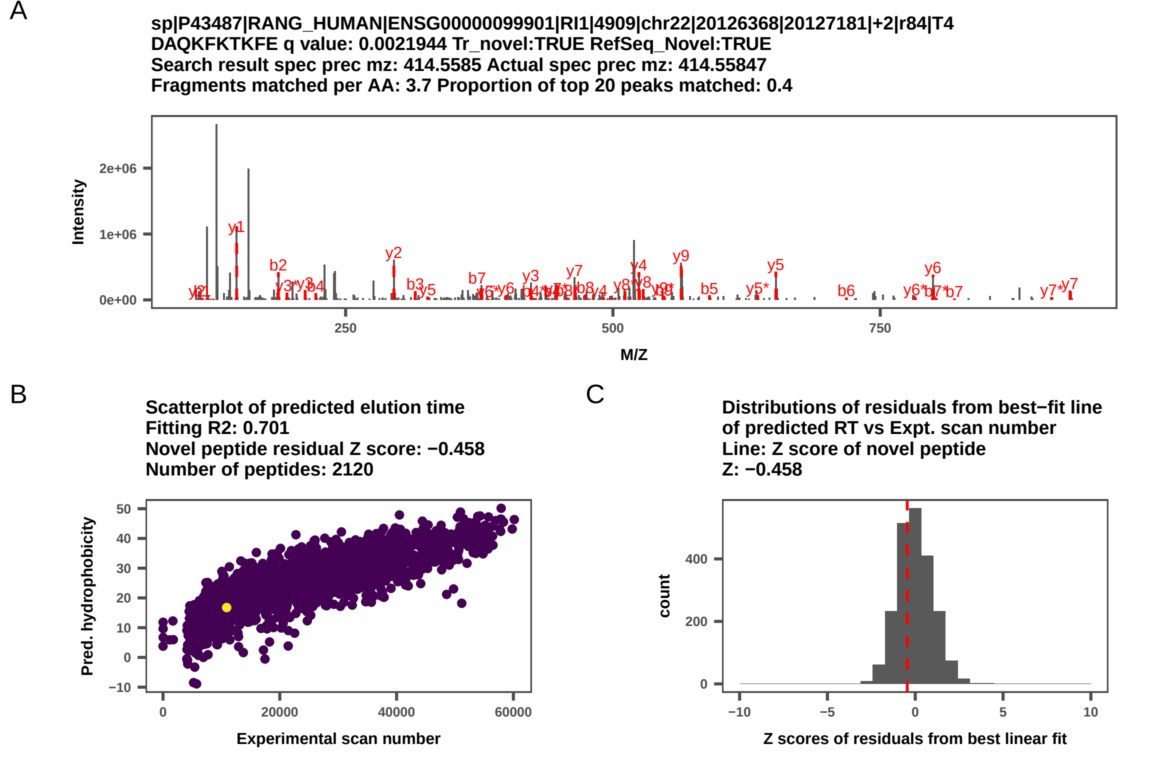
A
sp|P43487|RANG_HUMAN|ENSG00000099901|RI1|4909|chr22|20126368|20127181|+2|r84|T4
DAQKFKTKFE q value: 0.0021944 Tr_novel:TRUE RefSeq_Novel:TRUE
Search result spec prec mz: 414.5585 Actual spec prec mz: 414.55847
Fragments matched per AA: 3.7 Proportion of top 20 peaks matched: 0.4
Intensity
2e+06
1e+06
0e+00
y1
b2
y2
b3
y5
b7
y3
y7
y4
y9
y5
b5
y5*
b6
y6
b7
y7
250
500
750
M/Z
B
Scatterplot of predicted elution time
Fitting R2: 0.701
Novel peptide residual Z score: −0.458
Number of peptides: 2120
Pred. hydrophobicity
50
40
30
20
10
0
−10
0
20000
40000
60000
Experimental scan number
C
Distributions of residuals from best−fit line
of predicted RT vs Expt. scan number
Line: Z score of novel peptide
Z: −0.458
count
400
200
0
−10
−5
0
5
10
Z scores of residuals from best linear fit
